# The Role of Healthy Lifestyle in the Implementation of Regressing Suboptimal Health Status among College Students in China: A Nested Case-Control Study

**DOI:** 10.3390/ijerph14030240

**Published:** 2017-02-28

**Authors:** Jieyu Chen, Hongjie Xiang, Pingping Jiang, Lin Yu, Yuan Jing, Fei Li, Shengwei Wu, Xiuqiong Fu, Yanyan Liu, Hiuyee Kwan, Ren Luo, Xiaoshan Zhao, Xiaomin Sun

**Affiliations:** 1 School of Traditional Chinese Medicine, Southern Medical University, Guangzhou 510515, Guangdong, China; jieyu@smu.edu.cn (J.C.); 13427550499@163.com (P.J.); niuniu_jing@126.com (Y.J.); leephialf@126.com (F.L.); a2278197a@163.com (S.W.); siyecao2015@163.com (Y.L.); luoren2014@126.com (R.L.); 2 Department of Traditional Chinese Medicine, Shandong Provincial Qianfoshan Hospital, Shandong University, Jinan 250014, Shandong, China; xhjlyh@126.com; 3 Department of Traditional Chinese Medicine, The Affiliated Brain Hospital of Guangzhou Medical University (Guangzhou Huiai Hospital), Guangzhou 510170, Guangdong, China; yulinfimmu@126.com; 4 Centre for Cancer and Inflammation Research, School of Chinese Medicine, Hong Kong Baptist University, Hong Kong 999077, China; 13480405@life.hkbu.edu.hk (X.F.); hykwan@hkbu.edu.hk (H.K.)

**Keywords:** suboptimal health status (SHS), unhealthy lifestyle, SHS regression, health-promoting, nested case-control

## Abstract

*Background*: Suboptimal health status (SHS) is the intermediate health state between health and disease, it is medically undiagnosed and is also termed functional somatic syndrome. Although its clinical manifestations are complicated and various, SHS has not reached the disease status. Unhealthy lifestyle is associated with many chronic diseases and mortality. In accordance with the impact of lifestyle on health, it is intriguing to determine the association between unhealthy lifestyle and SHS risk. *Methods*: We conducted a nested case-control study among healthy Chinese college students from March 2012 to September 2013, which was nested in a prospective cohort of 5676 students. We performed 1:1 incidence density sampling with matched controls for birth year, sex, grade, specialty and individual character. SHS was evaluated using the medical examination report and Sub-health Measurement Scale V1.0 (SHMS V1.0). Exposure was defined as an unhealthy lifestyle per the frequency of six behavioral dimensions from the Health-promoting Lifestyle Profile (HPLP-II). *Results*: We matched 543 cases of SHS (42.66%) in a cohort of 1273 students during the 1.5 years mean follow-up time with controls. A significant difference (t = 9.79, *p* < 0.001) and a reduction in HPLP-II total score was present at 1.5 years follow-up (135.93 ± 17.65) compared to baseline (144.48 ± 18.66). A level-response effect was recorded with an increase of the total HPLP-II (every dimension was correlated with a decreased SHS risk). Compared to respondents with the least exposure (excellent level), those reporting a general HPLP-II level were approximately 2.3 times more likely to develop SHS (odd ratio = 2.333, 95% CI = 1.471 to 3.700); and those with less HPLP-II level (good level) were approximately 1.6 times more likely (1.644, 1.119–2.414) to develop SHS (*p* < 0.05). Our data indicated that unhealthy lifestyle behavior with respect to behavioral dimensions significantly affected SHS likelihood. Further analyses revealed a marked increase (average increased 14.73 points) in lifestyle level among those SHS regression to health after 1.5 years, with respect to the HPLP-II behavioral dimensions, in addition to the total score (t = −15.34, *p* < 0.001). *Conclusions*: SHS is highly attributable to unhealthy lifestyles, and the mitigation of modifiable lifestyle risk factors may lead to SHS regression. Increased efforts to modify unhealthy lifestyles are necessary to prevent SHS.

## 1. Background

In parallel with socio-economic development and increasing pace of life, there is a growing appreciation of the importance of health. The World Health Organization has defined health as “a state of complete physical, mental and social well-being and not merely the absence of disease or infirmity” [1]. Currently, the concept of health status is divided into three states: health, disease, and an intermediate state between health and disease, which we refer to as suboptimal health status (SHS). SHS is characterized by a decline in vitality, physiological function and the capacity for adaptation, and it is termed medically unexplained or functional somatic syndrome, which is common in Western countries [2,3,4,5]. People with SHS are always suffering from symptoms like chronic fatigue, headaches, dizziness, depression, anxiety, non-specific pain (such as back pain and chest pain, etc.), functional disorders of different organ systems (the digestive system, cardiovascular system, respiratory system, urinary system), which are linked to short-term or long-term adverse health outcomes. For instance, SHS markedly impairs quality of life, results in frequent hospital visits and large medical expenses, but no particular disease is evident. This is vexing and causes sufferers to become puzzled and disabled [3,4]. Our previous investigation also showed that SHS occurred in 46.0% of a surveyed population in Southern China [6] and 55.9% in Chinese students [5]. It becomes a growing health concern worldwide; multiple populated-based studies have shown an increase in the occurrence of SHS [3,7,8]. A recent China suboptimal health cohort study suggested that there may be a causative effect of SHS in non-communicable chronic diseases, and SHS may be an important status for the early detection and prevention of chronic disease [9]. However, both the etiology and occurrence of SHS, and the factors that promote its development are still largely unknown. There are two categories of health measures: subjective measures (questionnaire: e.g., 36-item Short-Form Health Survey Questionnaire (SF-36) [10], Suboptimal Health Status Questionnaire-25 (SHSQ-25) [11,12], Sub-Health Measurement Scale V1.0 (SHMS V1.0) [5,6]) and objective measures (anthological-physiological and biochemical measures, e.g., blood pressure (BP), body mass index (BMI), C-reactive protein (CRP), low density lipoprotein (LDL)) [13,14]. Currently, the main clinical diagnostics for SHS rely on the subjective and scale assessment after the comprehensive physical examination excluding specific disease, and the objective measurements for SHS are still being explored [15]. SHS questionnaires have been developed and are widely used as diagnostic instruments of SHS in China. For instance, SHSQ-25 that encompasses five subscales regarding fatigue, cardiovascular system, digestive tract, immune system and mental status, are mainly focused on physiological and psychological SHS [11,12]. SHMS V1.0, however, is a multidimensional, self-report symptom inventory including physiological, psychological and social dimensions [16], which is highly in accordance with the understanding of health in the WHO definition.

Unhealthy lifestyle is a well-known contributor to many health problems [17,18], and it is also a risk factor for cancer [19,20], diabetes mellitus [21,22], cardiovascular disease [23], obesity [24], metabolic syndrome [25], and irritable bowel syndrome [26]. Furthermore, lifestyle risk factors have been correlated with depression [27], fatigue, insomnia and nervousness [28], or even personal well-being [29]. Emerging evidence suggests that the increasing prevalence of SHS is related to lifestyle risk factors, such as poor dietary habits, sleep deprivation, violent social competition, sedentary activities, smoking and alcohol abuse [7,30,31,32]. However, results in these studies are confounded by a single lifestyle-related factor or retrospective exposure collection. Furthermore, this type of association could be explained by reversed causality or confounding from the cross-sectional investigation. College students receive a high level of education and begin to make most of their decisions without parental guidance, especially regarding health-promoting lifestyle choices and habits [33]. Students are absorbing an enormous amount of knowledge, facing fierce competition and challenges, so their health problems are of a great concern. To evaluate the nature of the association between unhealthy lifestyle-related factors with SHS risk, we conducted this study by using a prospective recording of exposure among college students in China. We hypothesized that there is a prospective association between lifestyle and SHS, healthy lifestyle and behaviors are jointly associated with a substantially lower risk of SHS, while the unhealthy lifestyle may lead to SHS.

## 2. Methods

### 2.1. Population Cohort

A prospective population cohort was established by consecutively enrolling 5987 college students who gave informed consent from March to May 2013. Two-thirds of the participants majored in medical subjects such as Clinical Medicine, Traditional Chinese Medicine, Integrated of Chinese Medicine and Western Medicine, Preventive Medicine, and Basic Medicine; and the other one-third in cross discipline subjects and multi-disciplinary fields related to medicine, such as Biometrical Engineering, Bioinformatics, Medical Laboratory Technology, Medical English, Medical Law, Health Economics, and Management of Public Health. Among the 5987 students, 311 students had spent their first two years on another campus, therefore they were excluded from the study and only 5676 students (1973 men and 3703 women) were included. These 5676 students were all studying on the same campus, which was a comfortable place for living and studying. Being on the same campus, they are presumed to be influenced by the same environmental factors and are provided the same facilities for their daily life.

The students underwent a health examination at the hospital. The health examination included medical history, physical examination, blood hematology and biochemistry analyses, resting electrocardiogram (ECG) and chest radiography. Baseline information was collected by a combination of self-designed questionnaire items and standardized questionnaires. The self-designed questionnaire items concerned general demographic characteristics, and the parts of the standardized questionnaires were taken from Sub-Health Measurement Scale V1.0 (SHMS V1.0) and Health-promoting Lifestyle Profile (HPLP-II) to assess the participants’ health status and lifestyle. After having the health examination and baseline assessment of the health status by SHMS V1.0, according to the clinical guidelines for SHS published by the China Association of Chinese Medicine (CACM) [5,15], all students were examined by our medical doctors for any diseases. After excluding participants diagnosed with clinical diseases as a result of the health examination, the threshold values for SHS in the physiological, psychological and society dimensions of SHMS V1.0 were 68, 67 and 67, respectively. If participants were not in SHS with respect to any of these three dimensions (physiological, psychological and society), they were considered healthy [5,34].

**Figure 1:**
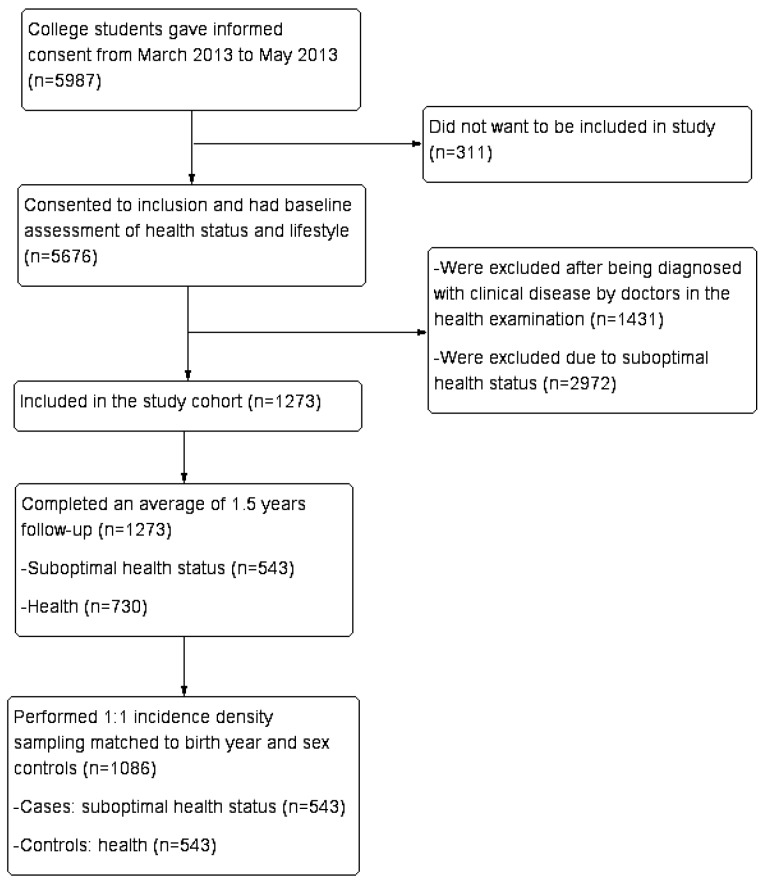
Flow of eligible participants in this nested case-control study.

For the study, 1273 healthy students and 4403 unhealthy students (1431 disease and 2972 SHS) were enrolled (Figure 1). Study cohort members entered the study on the date they completed the baseline questionnaires and they were followed until the end of September 2014. They were asked if they had experienced any uncomfortable symptoms every month. The same evaluation methods of health status (the physical examination and questionnaires) and lifestyle behaviors were repeated after 1.5 years.

### 2.2. Study Design

We carried out a case-control study nested in the baseline healthy students, 1273 health students (477 men and 796 women) aged 19.09 ± 1.08, which was nested in a prospective cohort of 5676 students. The flow of eligible participants in this nested case-control study is presented in Figure 1. We matched SHS (cases) in this cohort during the 1.5 years mean follow-up time with the healthy keeper (controls). Moreover, we performed 1:1 incidence density sampling with matched controls for birth year, sex, grade, specialty and individual character. Previous studies [5,6] indicated that men and women showed significant differences in lifestyle as well as in health status, so gender was also an important factor in our study for a matched analysis.

### 2.3. Definition and Diagnosis of Cases

Those students who had SHS at 1.5 years follow-up were considered as incident cases. To evaluate the robustness of SHS diagnosis, we relied on the medical examination report and SHMS V1.0. CACM characterized SHS as a decline in vitality, physiological function and the capacity for adaptation [5,32]. SHMS V1.0 was d ions of SHMS V1.0 were 68, 67 and 67, respectively [34]. A total of 543 cases of SHS were identified.

### 2.4. Selection of Control Subjects

Study cohort members who were still healthy at 1.5-year follow-up were eligible as control subjects. If participants did not have a clinical disease and were not rated unhealthy with respect to any of the three dimensions (physiological, psychological and social) of SHMS V1.0, they were considered healthy. Based on these criteria, we identified 730 controls. Additionally, we performed 1:1 incidence density sampling and matched controls by birth year, sex, grade, specialty and individual character; and 543 control subjects were randomly sampled and matched by SHS cases.

### 2.5. Exposure Assessment

For study purposes, we defined exposure as an unhealthy lifestyle. Lifestyle-related factors were assessed by the HPLP-II, which has been widely used as a measuring tool for assessing health-promoting behavior [35], and has been considered reliable and valid both domestically and internationally [36,37]. The HPLP-II is comprised of 52 items that assess six behavioral dimensions of lifestyle: spiritual growth, health responsibility, sports and exercise, nutrition, interpersonal relationships and stress management. This measuring instrument can be used to assess the frequency of health-promoting behavior using a self-reporting Likert scale, with a rating score ranging from 1 to 4 (never, sometimes, usually, always). The minimum and maximum HPLP-II scores were 52 and 208, respectively. A higher score represents a maximal level of health with respect to lifestyle. The health promoting lifestyle scores are divided into four levels; poor (52–90), general (91–129), good (130–168) and excellent (169–208).

### 2.6. Ethical Considerations

The study was approved by the Medical Ethics Committee of Nanfang Hospital in Guangzhou, China (2012) LunShenZi (Decision No. 035), and was conducted in accordance with approved guidelines and regulations. Written informed consents were obtained from subjects after the study objectives and methodology were clearly explained. These subjects were free to withdraw at any time without providing a reason. Strict confidentiality was maintained throughout the process of data collection and analysis.

### 2.7. Statistical Analysis

Descriptive statistics were presented as frequencies, means and standard deviations (SD). The primitive scores of health status assessment by SHMS V1.0 were converted to centesimal grades. Continuous variables (e.g., age, Body Mass Index (BMI), HPLP-II total score, SHMS V1.0 score) were analyzed with a *t*-test (for independent and paired samples). Dichotomous variables (e.g., smoking status, alcohol intake, level of HPLP) were analyzed with a χ^2^ test to determine significant differences. There were no significant differences in demographic variables between the case and control group, and without missing variables; so inclusion should not reduce the internal validity. We used *t*-test (for paired samples) to compare means between the self-reported discomfort assessment by SHMS V1.0 and the health-promoting lifestyle behaviors assessment by HPLP-II at baseline and at 1.5 years follow-up in cohort of college students participating in the study. We defined the reference group as those participants with the lowest level of exposure, i.e., excellent lifestyle behavior. Due to the category matching of the case-control study design, we used a conditional logistic regression analysis to estimate the odds ratios (OR) and 95% confidence intervals (CI) for the risk of SHS associated with health-promoting lifestyle behaviors according to the four-level scale, including the six dimensions of HPLP-II (spiritual growth, health responsibility, sports and exercise, nutrition, interpersonal relationships and stress management). The covariates were exposure to the lifestyle level of HPLP-II between the cases of SHS and their matched healthy controls. The analysis was repeated in a multivariate model which included the six dimensions of HPLP-II to estimate associations using health status as the outcomes of interest. All data analyses were done with SPSS 20.0. All *p* values were two-sided and we considered *p* < 0.05 as statistically significant.

## 3. Results

### 3.1. Baseline Characteristics of the Population Cohort and Case-Control Design

Different health status compared with both health–promoting lifestyle at the baseline of the population cohort is presented in Supplementary Figure S1 and Table S1. Students diagnosed with disease accounted for 25.2%, and SHS took up 52.4%. Analyses revealed significant differences with respect to six dimensions of health-promoting lifestyle and different health status, in addition to the total HPLP-II score (F (25676) = 584.11, *p* < 0.001). After Bonferroni correction for multiple comparisons, this difference was statistically significant (*p* < 0.001). The healthy group had score significantly higher on the total HPLP-II score and six dimensions, while the SHS group had the lowest, the disease group had a significantly lower score. The distribution of disease at baseline in the study cohort is showed in Supplementary Figure S2 and Table S2. The reported major diseases were related to the respiratory, digestive systems and endocrine or autoimmune systems, such as chronic rhinitis (47.43%), chronic pharyngolaryngitis (13.46%), chronic gastritis (12.86%), haemorrhoids (4.80%) and chronic insomnia (4.60%).

The cohort was comprised of 1273 healthy college students at the baseline that completed 1.5 years follow-up. We identified 543 cases with SHS, and matched 543 controls from the health keeper (Figure 1). Table 1 shows the baseline characteristics of the cases and controls at the time of recruitment. We found no significant differences in age, BMI, smoking status, and alcohol intake between the two groups at baseline. The overall level of HPLP-II was good, and there was a significant difference in the total specific HPLP-II score between the cases and controls (t = 5.017, *p* < 0.001).

### 3.2. Self-Reported Discomfort Assessment by SHMS V1.0 at Baseline and at 1.5 Years Follow-Up

Table 2 shows the self-reported discomfort assessment by SHMS V1.0 at the baseline and at 1.5 years follow-up. During a mean follow-up period of 1.5 years, 42.66% of healthy students developed a new case of SHS. There was a significant difference in health status changes in the cases during the year (t = 33.17, *p* < 0.001) according to the SHMS V1.0 total scores. Physiological dimension (physical condition, organ function, body movement function and vigor), physiological dimension (positive emotion, psychological symptoms and cognitive function), and social dimension levels (social adjustment, social resources and social support) were significantly lower at 1.5 years follow-up than at the baseline. However, neither significant differences in the SHMS V1.0 total score (t = −0.67, *p* = 0.506) nor other changes in the controls were noted, except that the levels of organ function (t = −2.17, *p* = 0.030) and social support (t = −2.02, *p* = 0.044) were significantly higher, and the level of positive emotion (t = 2.28, *p* = 0.023) was significantly lower after 1.5 years follow-up.

The primitive scores of health status assessment by SHMS V1.0 were converted to centesimal grades.

### 3.3. Health-Promoting Lifestyle Behavior Assessment by HPLP-II at Baseline and at 1.5 Years Follow-Up

Table 3 shows the health-promoting lifestyle behavior assessment by HPLP-II at baseline and at 1.5 years follow-up. There was a significant difference (t = 9.79, *p* < 0.001) and a marked reduction in the HPLP-II total score for the cases at 1.5 years follow-up (135.93 ± 17.65) compared to baseline (144.48 ± 18.66). For the six dimensions of HPLP-II, levels of spiritual growth, health responsibility, sports and exercise, interpersonal relationships and stress management were significantly lower at 1.5 years follow–up than at baseline, while the nutrition level was significantly higher. The controls also showed a significant difference (t = −3.86, *p* < 0.001) and an increase of the HPLP-II total score at 1.5 years follow-up (153.50 ± 18.42) compared to baseline (150.22 ± 19.01). Levels of sports and exercise, nutrition, and interpersonal relationships differed after 1.5 years follow-up and were significantly higher.

### 3.4. Association between Unhealthy Lifestyle with SHS Risk

Odd ratios pertaining to health-promoting lifestyle behaviors for college students that developed SHS compared to controls are presented in Table 4.

Respondents reporting a general level of HPLP-II were almost 2.3 times more likely to develop SHS (odd ratio (OR) = 2.333, 95% confidence interval (CI) = 1.471 to 3.700), while those less exposed (good level) were approximately 1.6 times more likely to develop SHS (OR = 1.644, 95% CI = 1.119 to 2.414), relative to those least exposed (excellent level) (*p* < 0.05). Results were consistent throughout the analysis, with all evidence indicating that unhealthy lifestyle behavior as indicated by the six HPLP-II dimensions had significant effects on the likelihood of SHS. For example, in the model adjusted for demographics variables, individuals with suboptimal spiritual growth (good), had a 50% or greater chance to develop SHS (1.550, 1.217–1.973) compared with those who showed an excellent level of spiritual growth; and individuals at the lowest level of health responsibility (poor) were approximately twice as likely to develop SHS (2.038, 1.113–3.733) than those at the excellent level. Odd ratios of 2.337 (1.328–4.114) and 3.650 (1.303–10.223) were associated with poor physical activity and poor nutrition, respectively, compared with those at an excellent level of the HPLP-II dimensions. Individuals who had a general level of interpersonal relationships were more than twice as likely to develop SHS 2.156 (1.133–4.104) , while odd ratios of 2.367 (1.302–4.302) were associated with a general level of stress management, compared to those at an excellent level.

### 3.5. The Health Status Evolution among 2972 SHS Students in 1.5 Years

We have further investigated the health status evolution through the study of the prospective population cohort among 2972 SHS students in 1.5 years, as shown in the Table 5. Table 5 shows the changes of 2972 SHS students after 1.5 years, which encompass 422 students with regression to health, 579 showing progression to disease, and 1971 maintaining SHS. Among those who displayed regression to health, the analyses revealed significant differences and a marked increase (average increased 14.73 points) in lifestyle level after 1.5 years compared to the baseline assessment, with respect to six dimensions of health–promoting lifestyle, in addition to the total HPLP-II score (t = −15.34, *p* < 0.001). Those who had disease, although the total HPLP-II score was improved (average increased 3.23 points), the five dimensions of HPLP-II were not statistically significant, including spiritual growth (t = 0.65, *p* = 0.516), health responsibility (t = −0.44, *p* = 0.660), sports and exercise (t = 1.69, *p* = 0.092), interpersonal relationship (t = −1.416, *p* = 0.157), and stress management (t = −1.25, *p* = 0.212). As for the ones who maintained their SHS status, the total HPLP-II scores appear to be increased to some extent (average increased 2.34 points). However, there is a non-significant trend to some dimensions of HPLP-II, including health responsibility (t = 1.64, *p* = 0.101) and interpersonal relationships (t = 1.24, *p* = 0.217).

## 4. Discussion

In this nested case-control study of college students, our analysis showed that an unhealthy lifestyle was associated with the incidence of SHS. A level-response effect with respect to the HPLP-II level was noted; an increased level for every behavioral dimension was correlated with a decreased risk of SHS. Mitigation of lifestyle risk factors might lead to the regression of SHS.

### 4.1. SHS and Prevention Medicine

SHS is an intermediate state between health and disease characterized by a low-quality condition of the mind and body, and Wang has proposed studying SHS to increase our understanding of health [38,39,40]. Though there is no definite physical diagnosis, SHS can be indicated by changes in physiology, psychology and social adaptation that lead to an inferior physiological state with regards to vitality, reaction ability and adaptability to the external environment. It has become a major public-health problem [3,5,6,8]. Evidence from the current study indicates that the incidence of SHS is high in college students; 42.66% of healthy students developed SHS during a mean 1.5 years follow-up period. Above all, a marked reduction was noted in every dimension and factor of the SHMS V1.0 score in the SHS group, while healthy individuals displayed an increase or no changes.

With SHS on the rise and sobering predictions for future adverse health effects as a result, prevention and intervention in SHS cases could be an important strategy and are therefore currently recommended. Traditional Chinese medicine has a similar role exemplified by the concept of preventive, predictive and personalized medicine, which is an effective approach for improving health in general and bringing SHS patients into a state of good health [38,39]. However, the mechanism underlying SHS has yet to be ascertained. Most current research [3,4,8,28,41,42] is focused on the complicated interactions of physical, chemical, and biological factors, as well as intrinsic factors of the body and psychological or socio-cultural factors. These factors can cause disharmony or threaten bodily homeostasis with functional disorders of the neuroendocrine immune network, oxidative stress injury, chronic psychosocial stress, a disturbance of energy metabolism, or the aberrant expression of many genes or proteins, which closely associated with a sustained somatic complaint. For instance, some researches have showed that SHS is associated with endothelial dysfunction. In addition, high SHS is associated with psychosocial stress, changes in cortisol level and/or glucocorticoid receptor isoform expression [13,14].

### 4.2. SHS and Unhealthy Lifestyle

Notably, findings emerging from several cross-sectional studies with large sample sizes have revealed overwhelming evidence that an unhealthy lifestyle is a risk factor for the occurrence and development of SHS [7,28,29]. However, little is known about how progressive changes in lifestyle–related factors that cause the deterioration of health status. Evidence of an association between a healthy lifestyle and the mitigation of SHS has arisen from cross-sectional studies that were not controlled for information bias, so the information from these studies might be obscured or elaborated resulting in relatively low confidence in such evidence. Our nested case-control analysis indicates that an unhealthy lifestyle can be considered as a SHS risk-equivalent, and poor health status can be reversed if the underlying cause of the health-related risk factors is addressed. Lifestyle factors affect health status in numerous ways, whether in physiological, psychological or social well-being.

Students who did not pay attention to their lifestyle were most likely to acquire SHS, while those who had a healthy lifestyle remained healthy, as reflected by a reduction of approximately 9 points in the average HPLP-II total score, as compared to the control that increased by nearly 4 points (*p* < 0.001). In a previous cross-sectional study, we found that five dimensions in the HPLP-II model, including spiritual growth, health responsibility, physical activity, interpersonal relations and stress management were related to SHS, but not nutrition [5]. More importantly, dynamic observation of the 1.5-year follow-up prospective cohort analyzed in this study showed that all six dimensions of the HPLP-II model were significantly and positively associated with health status, and that an unhealthy lifestyle was a risk factor for SHS. The current study revealed a marked association between the occurrence of SHS and poor lifestyle behavior and evidence of a positive frequency response between the likelihood of acquiring SHS and an unhealthy lifestyle, exemplified by the correlation of low HPLP-II scores with an increased likelihood of an adverse health response.

The HPLP-II framework encompassed self-reported health-promoting behaviors in six behavioral dimensions, underscoring the importance of the development of inner resources, one’s level of spiritual growth, maximizing human potential for wellbeing and fulfillment through a search for meaning, a sense of purpose, and working toward life goals [43]. The study participants were college students and their overall level of spiritual growth was relatively high. Individuals with suboptimal spiritual growth might have fewer inner resources, feel lonely, or lack a sense of meaning and life goals, and were more likely to exhibit SHS as reflected by a lower HPLP-II score. Human life is our highest value, and the concept of health is fundamental to life. Accordingly, health responsibility which includes focusing on one’s own health and educating oneself about health, is the basic requirement for the pursuit of happiness [44]. However, college students are relatively young and may not worry about their health status or notice transitory symptoms such as abdominal discomfort, dizziness, headaches, insomnia or fatigue, which may indicate the development of SHS due to poor health responsibility. Previous studies on the physical activity of students suggested that a majority were physically inactive or did not engage in habitual physical activity; meanwhile, insufficient physical activity was associated with weight gain, obesity, depression, anxiety disorders, sleep quality, or a low quality of life. Physical activity could reduce the risk of health problems by positively impacting self-awareness of physical fitness, self-esteem and better spiritual growth [45,46,47,48]. Our results not only augment previous findings that show a favorable independent effect of lifestyle factors on SHS among college students [5], but also confirm and extend the exact association between an unhealthy lifestyle and the risk of SHS, especially regarding the interactive effects of nutrition and SHS. Nutrition reflects the individual’s daily diet, which could affect physiological health. Studies have shown that many college students have poor dietary habits, such as skipping breakfast, and late-night and irregular eating that might cause poor nutritional status [49,50], and as a result, they may be at risk for SHS. Moreover, our previous study revealed that irregular breakfast eating habits were related to an increased risk of SHS and could be a useful predictor of an unhealthy lifestyle [6].

Since we are social beings shaped by experiences with others by a complex inherent motivation to interact with others, interpersonal relationships are important and critical to survival, adaptation, quality of life, and the pursuit of happiness. The inherent motivation to interact with others is complicated and different in different individuals and relationships are formed in the context of social, cultural and other influences. Interpersonal relationships have special significance for college students, especially because they lack varied and satisfactory interpersonal experience and are vulnerable to interpersonal relationships that directly affect their psychological and social health [51]. Moreover, coinciding with widespread computer use, some college students may become addicted to the internet by playing games, watching videos or surfing websites, all of which can result in poor interpersonal relationships and even affect academic achievement [52].

Internal and external demands can create stress for individuals, but appropriate pressure may provide high motivation to perform at one’s best. However, with stress increasing over time and a lack of proper management, a high stress level may have a negative effect on cognitive function and learning in students [53,54]. Our study showed that poor stress management may be a risk factor for SHS related to psychological, social, or even physiological health, exemplified by suffering from discomfort, depression, anxiety, mental illness, loneliness, compulsion, or even poor appetite, nervousness, palpitations, or sleeplessness. Previously, others have reported that college students experience greater stress, particularly academic stress, which contributes to the deterioration of the quality of life, and have poor interpersonal relationships with other students and faculty [55,56]; stress management might mitigate mental disorders, while physical activity and better spiritual growth conferred a lower perceived risk of stress [57]. Therefore, stress management has an important bearing on one’s general health and may be important to better health status.

### 4.3. Combination of Modifiable Risk Factors and the Implementation of SHS Regression

The proportion of SHS attributable to an unhealthy lifestyle was increased at a 1.5 years follow-up in college students. As SHS is an intermediate status in the transition between health and disease, SHS may be regression to health or progression to disease because of its bi-directional and translational nature, which can be substantiated by our results about the health status translation in Table 5, and the implementation of SHS regression would be expected to translate into better public health or clinical benefits. These findings emphasize the need for increased efforts to intervene in unhealthy lifestyles and to aggressively prevent and control SHS risk factors among those with an unhealthy lifestyle, especially young people. Our results showed that within the framework of the HPLP-II, the six behavioral dimensions of a health-promoting lifestyle were interconnected and a low total score had a strong relationship with the risk of SHS. Therefore, lifestyle intervention by mitigation of a combination of modifiable risk factors may be an effective way to promote health, including remaining healthy, or leading to the implementation of regressing SHS or disease. This finding is consistent with growing evidence that a healthy lifestyle reduces the risk of many chronic diseases or discomforts, such as certain types of cancers [58,59], coronary heart disease [60], diabetes [61], obesity [62], metabolic syndrome [63], irritable bowel syndrome [64], fatigue and mental disorders [65] and memory problems [66]. Furthermore, the results suggested the importance of more research in college students in order to identify the need for intervention and lifestyle improvement. Lifestyle counseling could be considered as an important part of health promotion and prevention programs. Favorable outcomes benefit not only individual students, but also potentially provide wide–spread benefits to other groups such as teachers and workers, thus emphasizing the need for further investigation of the association between lifestyle behaviors and the incidence of SHS.

### 4.4. Study Strengths and Limitations

The major strengths of our study were that the study design was a nested case-control study, with cohort data collected prospectively, using lifestyle information collected up to 1.5 years before SHS diagnosis, providing the opportunity to determine the temporal relationship between unhealthy lifestyle and subsequent development of SHS in college students. Additionally, compared with a cross-sectional or traditional case control design, a nested case–control analysis has superior computational efficiency for producing an odds ratio with minimized selection bias or recall bias. Furthermore, we studied the specific concept of SHS, including three dimensions and 10 factors related to physical, mental and social health, rather than an abstract concept. Last but not least, this study is the first study to demonstrate that the mitigation of a combination of modifiable lifestyle risk factors may lead to the regression of SHS.

The present study is observational and other potential confounding factors and biases could not be completely controlled. Data on unmeasured or unmeasurable risk factors such as family history were not available. The assessment of SHS must exclude disease and health, while any non-differential misclassification of these assessments would have biased the study results toward the null hypothesis and would not explain the strong associations and level-response relationships observed in this study. Because the sample size of healthy students was not large enough, the poor levels of the HPLP-II model were not statistically significant. Replicating these results in the context of a larger prospective cohort would be valuable. Future research should address these limitations.

## 5. Conclusions

In this nested case–control study of college students, our analysis showed that an unhealthy lifestyle is associated with the risk of SHS incidence in these students. A level-response effect was noted, with an increased level of HPLP-II and all behavioral dimensions correlated with a decreased risk of SHS. The proportion of SHS attributable to an unhealthy lifestyle increased after a mean 1.5 years follow-up SHS may be regression to health or progression to disease because of its bi–directional and translational nature. These findings emphasize the need for increased efforts to modify unhealthy lifestyles and to aggressively prevent and control SHS risk factors among those with an unhealthy lifestyle; accordingly, the mitigation of modifiable lifestyle risk factors may lead to SHS regression.

## Figures and Tables

**Table 1:** Baseline characteristics of cases and controls of study participants.

	Cases (n = 543)	Controls (n = 543)	Level of Significance
Men	188 (34.6%)	188 (34.6%)	-
Women	355 (65.4%)	355 (65.4%)	-
Mean (SD) age (years)	18.97 (1.07)	18.89 (1.06)	–1.28, * *p* = 0.200
BMI			
Baseline	19.99 (2.71)	20.21 (3.24)	1.257, * *p* = 0.209
Smoking status			
No	539 (99.3%)	539 (99.3%)	0, † *p* = 1.000
Yes	4 (0.7%)	4 (0.7%)	
Alcohol intake			
Never	279 (51.4%)	254 (46.8%)	4.14, † *p* = 0.247
Little	220 (40.5%)	240 (44.2%)	
Sometimes	44 (8.1%)	47 (8.7%)	
Often	0 (0)	2 (0.4%)	
Always	0 (0)	0 (0)	
HPLP-II			
Total score (0–208)	144.48 (18.66)	150.22 (19.01)	5.017, * *p* < 0.001
Level of HPLP	Good	Good	-

SHMS V1.0: Sub-Health Measurement Scale V1.0; HPLP-II: Health-promoting lifestyle profile; Data are n (%) or mean (SD). The primitive scores of health status assessment by SHMS V1.0 were converted to centesimal grades; * *t*-test for continuous variables; † χ^2^ for dichotomous variables.

**Table 2:** Self-reported discomfort assessment by SHMS V1.0 at baseline and at 1.5 years follow-up in cohort of college students participating in the study.

	Baseline	1.5 Years Follow-Up	Difference	Paired *t*-Test	*p*-Value
Cases					
SHMS V1.0 total score (0–100)	78.60 (4.70)	69.32 (5.45)	9.28 (6.52)	33.17	<0.001
Physiological dimension	80.63 (6.51)	74.97 (8.25)	5.65 (8.41)	15.68	<0.001
Physical condition	11.56 (1.74)	10.61 (1.94)	0.95 (2.15)	10.37	<0.001
Organ function	23.82 (2.49)	22.58 (2.94)	1.24 (3.01)	9.61	<0.001
Body movement function	14.68 (0.74)	14.25 (1.20)	0.43 (1.25)	7.96	<0.001
Vigour	9.09 (0.93)	8.55 (1.04)	0.55 (1.21)	10.48	<0.001
Physiological dimension	76.15 (6.06)	64.43 (7.92)	11.72 (8.84)	30.90	<0.001
Positive emotion	17.08 (1.57)	14.89 (2.05)	2.19 (2.29)	22.35	<0.001
Psychological symptoms	23.98 (2.08)	21.29 (2.54)	2.68 (2.74)	22.84	<0.001
Cognitive function	7.49 (1.00)	6.74 (1.05)	0.75 (1.10)	15.85	<0.001
Social dimension	78.70 (6.91)	67.05 (10.01)	11.65 (10.83)	25.08	<0.001
Social adjustment	16.49 (1.40)	14.83 (1.56)	1.66 (1.91)	20.26	<0.001
Social resources	12.75 (1.52)	10.69 (2.04)	2.06 (2.10)	22..87	<0.001
Social support	8.09 (0.95)	7.62 (1.18)	0.48 (1.26)	8.86	<0.001
Controls					
SHMS V1.0 total score (0–100)	80.80 (5.40)	80.97 (5.40)	–0.17 (6.10)	–0.67	0.506
Physiological dimension	82.87 (6.80)	83.32 (6.60)	–0.45 (0.32)	–1.392	0.165
Physical condition	11.96 (1.79)	11.93 (1.77)	0.03 (1.94)	0.35	0.724
Organ function	24.59 (2.42)	24.85 (2.35)	–2.56 (2.75)	–2.17	0.030
Body movement function	14.68 (0.73)	14.70 (0.69)	–0.02 (0.87)	–0.64	0.521
Vigour	9.18 (0.99)	9.18 (0.99)	0 (1.21)	–0.02	0.986
Physiological dimension	78.60 (6.83 )	78.60 (7.00)	0 (8.09)	0.01	0.996
Positive emotion	17.50 (1.62)	17.30 (1.70)	0.20 (2.01)	2.28	0.023
Psychological symptoms	24.53 (2.27)	24.70 (2.33)	–0.17 (2.58)	–1.54	0.123
Cognitive function	7.70 (1.01)	7.73 (0.95)	–0.02 (0.99)	–0.59	0.557
Social dimension	80.50 (7.19)	80.48 (7.40)	0.02 (8.30)	0.05	0.960
Social adjustment	16.77 (1.47)	16.77 (1.47)	0 (1.61)	0.03	0.979
Social resources	12.97 (1.53)	12.87 (1.53)	0.10 (1.66)	1.42	0.155
Social support	8.24 (0.94)	8.34 (0.96)	–0.10 (1.12)	–2.02	0.044

**Table 3:** Health-promoting lifestyle behaviors assessment by HPLP-II at baseline and at 1.5 years follow-up in cohort of college students participating in the study.

	Baseline	1.5 Years Follow-Up	Difference	Paired *t*-Test	*p*-Value
Cases					
HPLP-II total score (0–208)	144.48 (18.66)	135.93 (17.65)	8.55 (20.36)	9.79	<0.001
Spiritual growth	29.08 (3.92)	26.38 (4.09)	2.70 (4.39)	14.32	<0.001
Health responsibility	19.85 (4.67)	18.16 (4.14)	1.69 (5.01)	7.86	<0.001
Sports and exercise	19.46 (4.70)	18.11 (4.67)	1.35 (5.20)	6.08	<0.001
Nutrition	23.89 (4.50)	24.56 (4.26)	−0.66 (5.10)	−3.03	0.003
Interpersonal relationship	28.04 (3.55)	26.34 (3.86)	1.69 (4.27)	9.25	<0.001
Stress management	24.16 (3.48)	22.38 (3.36)	1.78 (3.94)	10.50	<0.001
Controls					
HPLP-II total score (0–208)	150.22 (19.01)	153.50 (18.42)	−3.28 (19.78)	−3.86	<0.001
Spiritual growth	30.04 (3.85)	30.05 (3.94)	−0.01 (4.35)	−0.06	0.949
Health responsibility	20.74 (5.16)	20.39 (4.99)	0.35 (5.48)	1.50	0.136
Sports and exercise	20.25 (4.88)	20.94 (5.04)	−0.69 (5.29)	–3.05	<0.001
Nutrition	25.10 (4.39)	27.13 (4.10)	−2.04 (4.82)	−9.85	<0.001
Interpersonal relationship	28.95 (3.62)	29.59 (3.59)	−0.64 (6.97)	−3.74	<0.001
Stress management	25.14 (3.42)	25.39 (3.45)	−0.25 (3.96)	−1.49	0.138

**Table 4:** Odd ratios and 95% confidence intervals for college students developing SHS group associated with the health-promoting lifestyle behaviors compared with the health group (OR = 1).

Dependent Variables	No (%) of Controls (n = 543)	No (%) of Cases (n = 543)	OR (95% CI)	*p*-Value
Health-promoting lifestyle				
Poor	0	1	-	-
General	79	111	2.333 (1.471–3.700)	<0.001
Good	378	377	1.644 (1.119–2.414)	0.011
Excellent	86	54	Reference	
Spiritual growth				
Poor	0	1	-	-
General	14	15	1.309(0.608–2.819)	0.491
Good	226	285	1.550(1.217–1.973)	<0.001
Excellent	303	242	Reference	
Health responsibility				
Poor	71	89	2.038 (1.113–3.733)	0.021
General	303	335	1.775 (1.028–3.064)	0.039
Good	132	96	1.148 (0.643–2.050)	0.640
Excellent	37	23	Reference	
Sports and exercise				
Poor	33	47	2.337 (1.328–4.114)	0.003
General	239	245	1.678 (1.139–2.471)	0.009
Good	181	194	1.736 (1.168–2.579)	0.006
Excellent	90	57	Reference	
Nutrition				
Poor	6	14	3.650 (1.303–10.223)	0.014
General	146	197	2.040 (1.348–3.087)	0.001
Good	307	272	1.289 (0.883–1.881)	0.188
Excellent	84	60	Reference	
Interpersonal relationship				
Poor	0	1	-	-
General	18	27	2.156 (1.133–4.104)	0.019
Good	285	334	1.599 (1.236–2.068)	<0.001
Excellent	240	181	Reference	
Stress management				
Poor	1	2	3.095 (0.277–34.601)	0.359
General	22	37	2.367 (1.302–4.302)	0.005
Good	279	322	1.547 (1.197–2.000)	0.001
Excellent	241	182	Reference	

**Table 5:** The health status translation and the assessment of lifestyle behaviors through the study of the prospective population cohort among 2972 SHS students in 1.5 years follow-up.

	Baseline	1.5 Years Follow-Up	Difference	Paired *t*-Test	*p*-Value
SHS→Health (n = 422)					
HPLP-II total score (0–208)	132.72 (16.56)	147.45 (18.76)	–14.73 (19.73)	–15.34	<0.001
Spiritual growth	26.33 (3.99)	28.87 (4.04)	–2.54 (4.49)	–11.631	<0.001
Health responsibility	18.16 (3.93)	19.71 (5.00)	–1.55 (5.39)	–5.902	<0.001
Sports and exercise	17.42 (4.37)	19.78 (5.14)	–2.37 (5.26)	–9.234	<0.001
Nutrition	22.68 (4.19)	25.94 (3.95)	–3.27 (4.68)	–14.332	<0.001
Interpersonal relationship	25.92 (3.56)	28.57 (3.76)	–2.66 (4.04)	–13.50	<0.001
Stress management	22.22(3.20)	24.57(3.28)	–2.35(3.93)	–12.282	<0.001
SHS→SHS (n = 1971)					
HPLP-II total score (0–208)	126.00 (16.37)	128.34 (17.52)	–2.34 (17.23)	–6.04	<0.001
Spiritual growth	24.82 (4.36)	24.41 (4.37)	0.41 (4.47)	4.07	<0.001
Health responsibility	17.04 (3.68)	16.80 (3.97)	0.15 (4.19)	1.64	0.101
Sports and exercise	16.52 (4.05)	17.20 (4.53)	–0.68 (4.64)	–6.49	<0.001
Nutrition	21.82 (4.09)	23.72 (4.07)	–1.89 (4.60)	–18.28	<0.001
Interpersonal relationship	24.69 (3.74)	24.81 (3.90)	–0.11 (4.01)	–1.24	0.217
Stress management	21.10 (3.25)	21.33 (3.42)	–0.22 (3.68)	–2.71	0.007
SHS→Disease (n = 579)					
HPLP-II total score (0–208)	126.40 (17.79)	129.63 (18.94)	–3.23 (18.82)	–4.13	<0.001
Spiritual growth	24.76 (4.52)	24.63 (4.82)	0.13 (4.79)	0.65	0.516
Health responsibility	17.31 (3.82)	17.40 (4.22)	–0.08 (4.58)	–0.44	0.660
Sports and exercise	16.84 (4.23)	17.20 (4.64)	–0.35 (5.04)	–1.69	0.092
Nutrition	21.77 (4.33)	24.25 (4.26)	–2.48 (4.59)	–12.98	<0.001
Interpersonal relationship	24.74 (3.94)	24.99( 4.42)	–0.25 (4.30)	–1.416	0.157
Stress management	20.97 (3.58)	21.17(3.66)	–0.19 (3.74)	–1.25	0.212

## Supplementary Materials

The following are available online at www.mdpi.com/1660-4601/14/3/240/s1. Figure S1. The health-promoting lifestyle (HPLP-II) total score of different health status. Table S1. Health status compared with health–promoting lifestyle using one-way ANOVA. Figure S2. Distribution of disease at baseline in the study cohort. Table S2. Distribution of disease at baseline in the study cohort (n = 1431).
