# Supplementary material for: The Role of Healthy Lifestyle in the Implementation of Regressing Suboptimal Health Status among College Students in China: A Nested Case-Control Study
Source: Int J Environ Res Public Health. 2017 Feb 28;14(3):240. doi: 10.3390/ijerph14030240 (PMC5369076; doi:10.3390/ijerph14030240)
Supplement: Supplementary file 1 [file ijerph-14-00240-s001.pdf]

Supplementary Materials: The Role of Healthy Lifestyle in the Implementation of Regressing Suboptimal Health Status among College Students in China: A Nested Case–Control Study

Jieyu Chen, Hongjie Xiang, Pingping Jiang, Lin Yu, Yuan Jing, Fei Li, Shengwei Wu, Xiuqiong Fu, Yanyan Liu, Hiuyee Kwan, Ren Luo, Xiaoshan Zhao and Xiaomin Sun

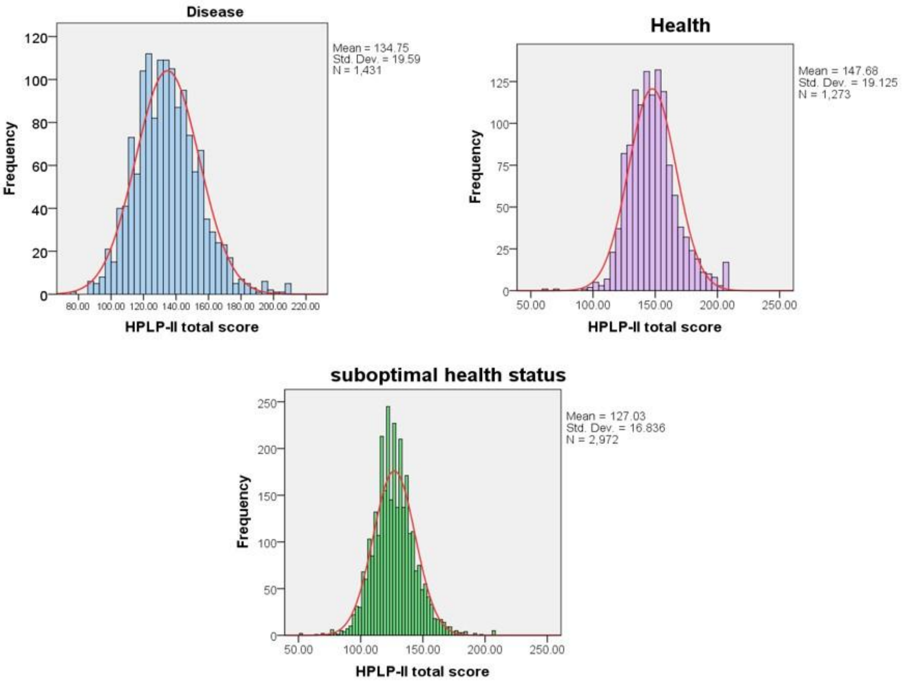

Figure S1. The health-promoting lifestyle (HPLP-II) total score of different health status.

Table S1. Health status compared with health-promoting lifestyle using one-way ANOVA.

	**Health status**			**One-way ANOVA**		
	**Group 1: Health (n = 1273, 22.4%)**	**Group 2: SHS (n = 2972, 52.4%)**	**Group 3: Disease (n = 1431.25.2%)**	**F value**	***p*-value**	**Multiple comparisons**
Spiritual growth	29.58 (3.90)	25.02(4.37)	26.76 (4.86)	480.10	0.000	G3 < G2 < G1 *
Health responsibility	20.37 (4.95)	17.25 (3.76)	18.39 (4.37)	245.08	0.000	G2 < G3 < G1 *
Sports and exercise	19.92 (4.81)	16.71 (4.14)	17.51 (4.47)	240.61	0.000	G2 < G3 < G1 *
Nutrition	24.64 (4.50)	21.93 (4.16)	23.04 (4.36)	179.46	0.000	G2 < G3 < G1 *
Interpersonal relationship	28.51 (3.62)	24.88 (3.78)	26.54 (4.14)	410.75	0.000	G2 < G3 < G1*
Stress management	24.67 (3.49)	21.24 (3.36)	22.49 (3.80)	432.71	0.000	G2 < G3 < G1 *
Total score	147.68 (19.13)	127.03 (16.84)	134.75 (19.60)	584.11	0.000	G2 < G3 < G1 *

Data presented as mean (SD). ANOVA indicates analysis of variance. Bonferroni was used in the multiple comparisons; *p <0.001 (Significant after Bonferroni correction for post-hoc analysis.)

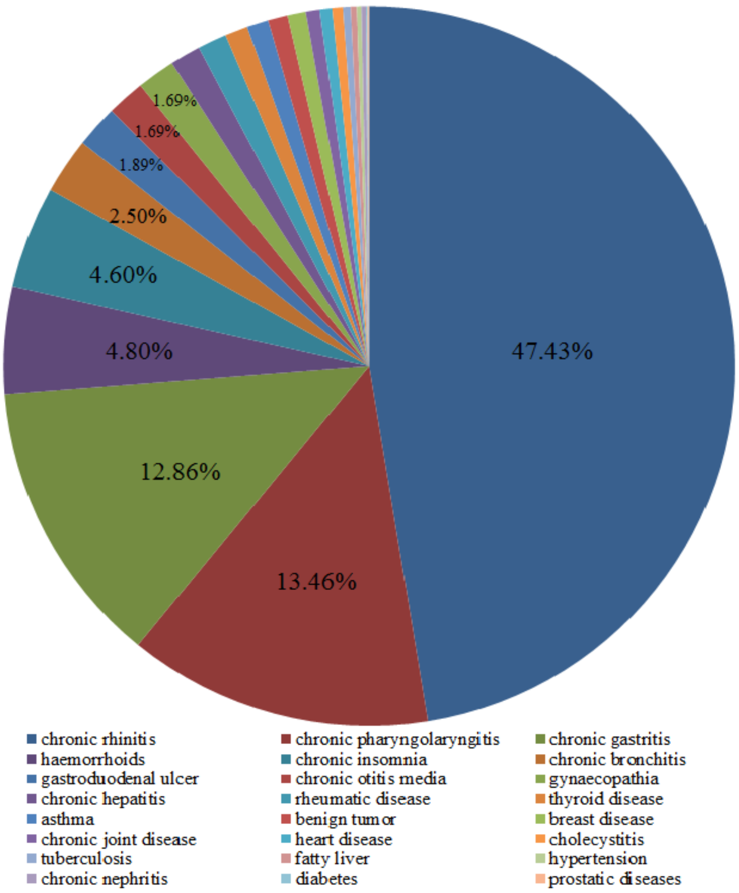

Figure S2. Distribution of disease at baseline in the study cohort (n = 1431). The reported major diseases were related to the respiratory, digestive systems and endocrine or autoimmune systems, such as chronic rhinitis (47.43%), chronic pharyngolaryngitis (13.46%), chronic gastritis (12.86%), haemorrhoids (4.80%) and chronic insomnia (4.60%).

Table S2. Distribution of disease at baseline in the study cohort (n = 1431).

Disease	Count	Percentage
chronic rhinitis	701	47.43%
chronic pharyngolaryngitis	199	13.46%
chronic gastritis	190	12.86%
haemorrhoids	71	4.80%
chronic insomnia	68	4.60%
chronic bronchitis	37	2.50%
gastroduodenal ulcer	28	1.89%
chronic otitis media	25	1.69%
gynaecopathia	25	1.69%
chronic hepatitis	20	1.35%
rheumatic disease	19	1.29%
thyroid disease	15	1.01%
asthma	14	0.95%
benign tumor	13	0.88%
breast disease	12	0.82%
chronic joint disease	9	0.61%
heart disease	8	0.54%
cholecystitis	7	0.47%
tuberculosis	5	0.34%
fatty liver	4	0.27%
hypertension	3	0.20%
chronic nephritis	3	0.20%
diabetes	1	0.07%
prostatic diseases	1	0.07%

© 2017 by the authors. Submitted for possible open access publication under the terms and conditions of the Creative Commons Attribution (CC–BY) license (http://creativecommons.org/licenses/by/4.0/).
