# Supplementary material for: The Role of Healthy Lifestyle in the Implementation of Regressing Suboptimal Health Status among College Students in China: A Nested Case-Control Study
Source: Int J Environ Res Public Health. 2017 Feb 28;14(3):240. doi: 10.3390/ijerph14030240 (PMC5369076; doi:10.3390/ijerph14030240)
Supplement: Supplementary file 2 [file ijerph-14-00240-s002.docx]

Supplementary Materials: The Role of Healthy Lifestyle in the Implementation of Regressing Suboptimal Health Status among College Students in China: A Nested Case–Control Study

Jieyu Chen, Hongjie Xiang, Pingping Jiang, Lin Yu, Yuan Jing, Fei Li, Shengwei Wu, Xiuqiong Fu, Yanyan Liu, Hiuyee Kwan, Ren Luo, Xiaoshan Zhao and Xiaomin Sun

**
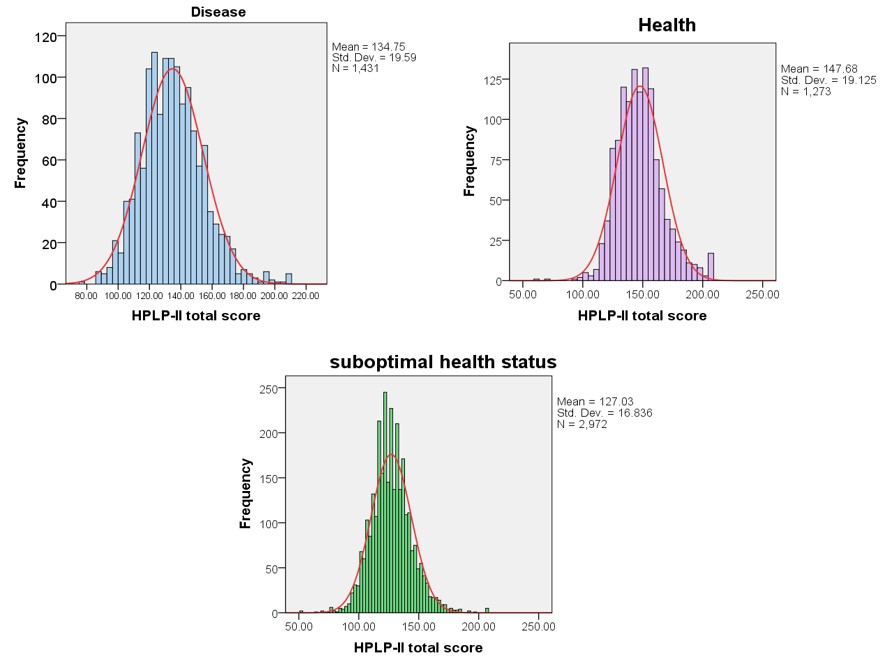
**

**Figure S1.** The health-promoting lifestyle (HPLP-II) total score of different health status.

**Table S1.** Health status compared with health-promoting lifestyle using one-way ANOVA.

|  | Health status | | | One-way ANOVA | | |
| --- | --- | --- | --- | --- | --- | --- |
|  | Group 1: Health (n = 1273, 22.4%) | Group 2: SHS (n = 2972, 52.4%) | Group 3: Disease (n = 1431.25.2% ) | F value | *p*-value | Multiple comparisons |
| Spiritual growth | 29.58 (3.90) | 25.02(4.37) | 26.76 (4.86) | 480.10 | 0.000 | G3 < G2 < G1 * |
| Health responsibility | 20.37 (4.95) | 17.25 (3.76) | 18.39 (4.37) | 245.08 | 0.000 | G2 < G3 < G1 * |
| Sports and exercise | 19.92 (4.81) | 16.71 (4.14) | 17.51 (4.47) | 240.61 | 0.000 | G2 < G3 < G1 * |
| Nutrition | 24.64 (4.50) | 21.93 (4.16) | 23.04 (4.36) | 179.46 | 0.000 | G2 < G3 < G1 * |
| Interpersonal relationship | 28.51 (3.62) | 24.88 (3.78) | 26.54 (4.14) | 410.75 | 0.000 | G2 < G3 < G1* |
| Stress management | 24.67 (3.49) | 21.24 (3.36) | 22.49 (3.80) | 432.71 | 0.000 | G2 < G3 < G1 * |
| Total score | 147.68 (19.13) | 127.03 (16.84) | 134.75 (19.60) | 584.11 | 0.000 | G2 < G3 < G1 * |

Data presented as mean (SD). ANOVA indicates analysis of variance. Bonferroni was used in the multiple comparisons; *p <0.001 (Significant after Bonferroni correction for post-hoc analysis.)


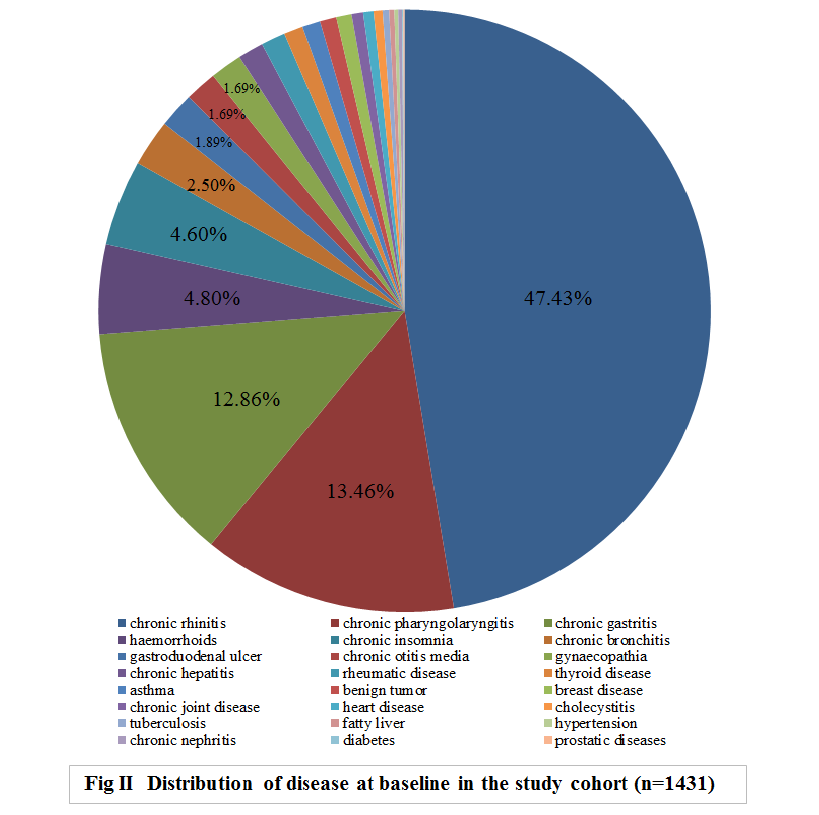


**Figure S2.** Distribution of disease at baseline in the study cohort (n = 1431). The reported major diseases were related to the respiratory, digestive systems and endocrine or autoimmune systems, such as chronic rhinitis (47.43%), chronic pharyngolaryngitis (13.46%), chronic gastritis (12.86%), haemorrhoids (4.80%) and chronic insomnia (4.60%).

**Table S2.** Distribution of disease at baseline in the study cohort (n = 1431).

| **Disease** | **Count** | **Percentage** |
| --- | --- | --- |
| chronic rhinitis | 701 | 47.43% |
| chronic pharyngolaryngitis | 199 | 13.46% |
| chronic gastritis | 190 | 12.86% |
| haemorrhoids | 71 | 4.80% |
| chronic insomnia | 68 | 4.60% |
| chronic bronchitis | 37 | 2.50% |
| gastroduodenal ulcer | 28 | 1.89% |
| chronic otitis media | 25 | 1.69% |
| gynaecopathia | 25 | 1.69% |
| chronic hepatitis | 20 | 1.35% |
| rheumatic disease | 19 | 1.29% |
| thyroid disease | 15 | 1.01% |
| asthma | 14 | 0.95% |
| benign tumor | 13 | 0.88% |
| breast disease | 12 | 0.82% |
| chronic joint disease | 9 | 0.61% |
| heart disease | 8 | 0.54% |
| cholecystitis | 7 | 0.47% |
| tuberculosis | 5 | 0.34% |
| fatty liver | 4 | 0.27% |
| hypertension | 3 | 0.20% |
| chronic nephritis | 3 | 0.20% |
| diabetes | 1 | 0.07% |
| prostatic diseases | 1 | 0.07% |

© 2017 by the authors. Submitted for possible open access publication under the terms and conditions of the Creative Commons Attribution (CC–BY) license (http://creativecommons.org/licenses/by/4.0/).
